# Surface Modification of Biomedical Ti-18Zr-15Nb Alloy by Atomic Layer Deposition and Ag Nanoparticles Decoration

**DOI:** 10.3390/jfb14050249

**Published:** 2023-04-28

**Authors:** Anton Konopatsky, Tatyana Teplyakova, Vadim Sheremetyev, Tamara Yakimova, Olga Boychenko, Marina Kozik, Dmitry Shtansky, Sergey Prokoshkin

**Affiliations:** 1 National University of Science and Technology “MISIS”, Leninsky Prospect 4s1, 119049 Moscow, Russia; 2 A.V. Shubnikov Institute of Crystallography, FSRC “Crystallography and Photonics” RAS, 119333 Moscow, Russia; 3 School of Chemistry, Lomonosov Moscow State University, 119991 Moscow, Russia

**Keywords:** superelastic Ti-Zr-Nb alloy, surface modification, atomic layer deposition, Ag nanoparticles synthesis, antibacterial properties

## Abstract

Superelastic biocompatible alloys attract significant attention as novel materials for bone tissue replacement. These alloys are often composed of three or more components that lead to the formation of complex oxide films on their surfaces. For practical use, it is desirable to have a single-component oxide film with a controlled thickness on the surface of biocompatible material. Herein we investigate the applicability of the atomic layer deposition (ALD) technique for surface modification of Ti-18Zr-15Nb alloy with TiO_2_ oxide. It was found that a 10–15 nm thick, low-crystalline TiO_2_ oxide layer is formed by ALD method over the natural oxide film (~5 nm) of the Ti-18Zr-15Nb alloy. This surface consists of TiO_2_ exclusively without any additions of Zr or Nb oxides/suboxides. Further, the obtained coating is modified by Ag nanoparticles (NPs) with a surface concentration up to 1.6% in order to increase the material’s antibacterial activity. The resulting surface exhibits enhanced antibacterial activity with an inhibition rate of more than 75% against *E. coli* bacteria.

## 1. Introduction

The growing demand for improving quality of life, as well as the aging of the population, has led to the intensive development of novel metallic biomaterials for implants in orthopedics, maxillofacial surgery and dentistry [1,2]. Many new alloys, methods of their processing and surface modifications have been developed in the last decade to improve the biological and mechanical compatibility of metallic biomaterials [1,2,3,4,5,6]. One of the most important tasks in this context is the selection and adequate combination of the alloy composition, its processing and surface treatment methods to promote bio-functionalization to a qualitatively new level.

Titanium alloys are the most commonly used among metallic biomaterials due to their unique combination of high strength, relatively low Young’s modulus and high corrosion resistance [2,7,8]. However, the problem of their biomechanical compatibility with bone tissue remains far from resolution. The difference between the Young’s modulus of the bone (0.1–30 GPa) and the implant material (112 GPa for Ti-6Al-4V [2]) provokes the development of the “stress shielding” phenomenon and subsequent resorption of the bone tissue around the implant [9]. Therefore, metastable β-titanium shape-memory alloys (SMA) exhibiting a low Young’s modulus (40–60 GPa) have attracted increased attention in recent years [1,2,10,11,12,13,14,15]. Among these SMAs, the Ti-18Zr-15Nb (at.%) alloy is the most promising due to its excellent superelasticity at human body temperature and large theoretical superelastic recovery strain (5–6%) [16,17,18]. The development of new approaches to surface modification of such alloys is a relevant issue.

So far, many contemporary surface modification methods have been applied to enhance the biological activity of T-Zr-Nb-based SMAs [19,20,21,22,23,24,25,26,27,28]. Most of them, such as anodization [19,21], plasma electrolytic oxidation [24] and thermal oxidation [25,26], are related to the formation of a surface layer based on the oxides of the main components of the alloy: TiO_2_, NbO_2_, Nb_2_O_5_ and ZrO_2_. In some cases, it is also possible to form more complex oxides containing two main alloy components such as TiNbO_4_ and TiNb_2_O_7_ [25,26]. It is well known that each of these compounds has high biocompatibility [25] and TiO_2_-based oxide surfaces can also demonstrate high biological activity and excellent blood compatibility [29,30]. The multicomponent nature of the Ti-Zr-Nb alloy imposes significant restrictions on the controlled modification of its surface. Ti, Nb and Zr oxides have differing chemical stability, hence they act differently when exposed to chemical etching, which is a promising and simple method of surface modification [31]. In this case, the formation of a single-component titanium oxide film on the surface of the alloy appears to be beneficial for its further modification. 

Atomic layer deposition (ALD) is a long-known and widely-used method for the controlled formation of thin TiO_2_-based films on the surface of titanium alloys [32,33,34,35,36,37]. The thickness of the TiO_2_ coatings can be controlled by the number of alternating reaction cycles in the range from 3 to 100 nm [34]. It was demonstrated that biological activity depends on the coating thickness and crystallinity, whose optimum values were obtained at 300 deposition cycles [35]. It was shown that TiO_2_ anatase surface layers successfully deposited by ALD exhibit improved surface hydrophilicity [38] and enhancement in osteoblast adhesion, spreading and growth [33,39]. The results of in vivo experiments have shown a significant advantage in the osseointegration ability of ALD TiO_2_-coated dental implants compared to their uncoated counterparts [40]. The osteopromotive properties of the ALD coating can be further improved by collagen functionalization, which leads to accelerated healing of bone defects [41]. Thus, the use of the ALD technique for the surface modification of multicomponent Ti-Zr-Nb SMAs seems promising due to the formation of a single-component (TiO_2_ anatase), thin and bioactive surface layer.

In addition to biological activity, the functional characteristics of the implant are affected by the number of bacterial colonies at the implant–bone interface [42]. Emerging bacterial inflammation can lead to serious damage of both soft tissues and bones [43]. Therefore, there is a need to limit such inflammation and increase the antibacterial properties of an implant. Various silver modifications have been known for their bactericidal properties for many years [44], but nanoscale particles have attracted special attention only in recent years [45]. The use of Ag NPs is more efficient than metallic silver coatings and their compounds [46]. The key factor determining antibacterial activity is the release rate of Ag ions [47]. Ag NPs are characterized by a high specific surface area, which makes it possible to tune the ion release with a high accuracy. Titanium discs with Ag NPs and Ag content up to 2.7 at.%, obtained by direct current sputtering, showed good antibacterial properties against *S. mutans* and *P. aeruginosa* while maintaining high fibroblast cytocompatibility [48]. Ag NPs embedded in a thin organosilicon film by plasma deposition at atmospheric pressure in a loading dose of 15.4 at.% completely suppressed the growth of *E. coli* strain [49]. TiO_2_/Ag NPs nanocomposite obtained using the two-step ALD method demonstrated a stable antibacterial effect against *S. aureus* and good affinity for MG-63 osteoblast-like cells [50]. Ion-implanted silver significantly increased the bactericidal properties of ZrO_2_ against oral microorganisms, despite the low ion release rate (below 1 ppm after a month of exposure) and low cytotoxicity against gingival fibroblasts [51]. Thus, it can be concluded that, under carefully-selected synthesis parameters, Ag NPs appear to be a promising antibacterial agent [52].

In this work, a combination of the ALD method for the formation of a thin TiO_2_ oxide layer on the surface of a superelastic Ti-18Zr-15Nb alloy and the subsequent synthesis of Ag NPs was implemented for the first time. The developed approach made it possible to precisely control the chemical composition of the surface oxide film and significantly improve its antibacterial properties through the adding of a bactericidal component. Special attention is paid to the coating cross-section characterization by transmission electron microscopy (TEM) and in-depth elemental profiling by the X-ray photoelectron spectroscopy (XPS) technique. 

## 2. Materials and Methods

### 2.1. Coating Formation

Disks of Ti-18Zr-15Nb alloy with a diameter of 10 mm and a thickness of 3 mm were used as the substrates. The alloy surfaces were prepared using an ATM “Saphir 560” (Blieskastel, Germany) grinding and polishing machine. Firstly, the samples were mechanically wet-ground using emery paper up to #4000. Next, they were chemically-mechanically polished on cloth to reach “optical brightness”, using a 0.05 μm fraction of SiO_2_, hydrogen peroxide 3%, and ammonia.

The ALD technique for TiO_2_ thin films deposition was realized according to [34,35] using a hot-wall low-pressure (1.5 mbar) Sunale R-150 Picosun OY ALD reactor (Masala, Finland). Titanium ethoxide Ti(OC_2_H_5_)_4_ at 150 °C and water H_2_O at 22 °C were applied for the TiO_2_ film’s growth. One reaction cycle was carried out in the following order: titanium ethoxide pulse (0.1 s), purging pulse (4.0 s), water pulse (0.2 s), and purging pulse (4.0 s). The reaction cycle was repeated 300 times, according to the regime described elsewhere [35]. The temperature of the substrate was kept at 250 °C. Nitrogen with a purity of 99.999% was used as a carrier and purging gas.

### 2.2. Ag NPs Synthesis

The synthesis method is based on the silver borohydride reduction [53]. For the first series of samples, the following synthesis technique was used. NaBH_4_ solution (concentration of 0.076 mg/mL) was cooled down to 1 °C, then 10 mL of AgNO_3_ solution (0.255 mg/mL) was added dropwise over a period of 10 min. The solution color was bright yellow, indicating the formation of colloidal Ag NPs. The ALD sample was submerged into the obtained solution for 12 h under mild stirring conditions. After that, the sample was dried under the hood overnight (designated as Ag/ALD). For the second series of samples, the technique was modified as follows: a droplet of NaBH_4_ solution (2 mg/mL) was placed on the surface of the ALD sample and dried at ambient conditions. Then the sample was submerged into 50 mL AgNO_3_ solution (1 mg/mL) for 20 min, washed with distilled water and dried overnight. These samples were designated as Ag(d)/ALD.

### 2.3. Characterization

The near-surface layer phase composition was studied using X-ray diffraction on an Ultima IV difractometer (Rigaku, Tokyo, Japan) with CuK_α_ radiation with parallel beam and graphite monochromators in the 20 to 80 deg 2θ range. X-ray surveying was carried out in asymmetric geometry with a constant angle of incidence of the primary beam of 5 degrees according to the technique described elsewhere [54]. Surface structure was analyzed by scanning electron microscopy (SEM), using a JEOL 7600F instrument equipped with an energy dispersive X-ray spectroscopy (EDX) analyzer (Oxford Instruments) at 15 kV accelerating voltage. Electron images were obtained in secondary electrons (SEI), as well as higher energy backscattered electrons (BSE). The specimen cross-section was prepared using a focused ion beam (30 kV, Ga+ ions, tilt of 52°) on a Scios dual beam (FEI). Pt alloy was applied before cross-sectioning to prevent unwanted specimen etching. Transmission electron microscopy (TEM) and energy dispersive X-ray spectroscopy (EDS) were conducted using a Tecnai Osiris 200 kV electron microscope (FEI, Hillsboro, OR, USA) with a point resolution of 2.5 Å, equipped with a high-angle annular dark field (HAADF) detector (FEI, Hillsboro, OR, USA). Image processing was carried out using the ImageJ software (1.53t version).

Surface chemical state was studied using the X-ray photoelectron spectroscopy (XPS) method on a PHI 5500 VersaProbeII (Chanhassen, MI, USA) unit. Depth profiling was conducted by 2 keV Ar^+^ sputtering with a standard sputtering rate of 16 nm/min (for SiO_2_). The TiO_2_ sputtering rate is calculated using a 0.54 coefficient in regard to SiO_2_ model substrate [55]. Spectrum approximation was conducted using the CasaXPS software (2.3.25 version) and Shirley background. For calibration, the C1s peak position was set at 285.0 eV. Ag NP dissolution and corresponding Ag^+^ ion release were studied by inductively coupled plasma (ICP) emission spectrometry on a iCAP 6300 instrument (Thermo Fisher Scientific, Waltham, MA, USA). Ag NP-decorated samples were immersed in 50 mL of distilled water and left on a rotary table for 24 h. After that, 10 mL probes were collected and analyzed.

### 2.4. Antibacterial Tests

A wild *E. coli* strain with a concentration of 1 × 10^9^ CFU/mL was cultivated in liquid medium before testing. Two LB media favoring bacteria growth were prepared: liquid and solid. For the liquid LB medium with a neutral pH of 7.4, meat peptone (Lot: BCBR6120V), yeast (Lot: SLBR0580V) and NaCl (Sigma Aldrich, St. Louis, MO, USA) were used; for the solid LB medium, agar (Bacto, Lot: 2299369) was also added.

ALD, Ag/ALD and Ag(d)/ALD samples were placed in 24-well plates and 100 μL of *E. coli* suspension in LB broth was added on their surface (approx. 1 × 10^2^ CFU/disk). In order to maintain humidity in the plate, a small amount of distilled water was added into the plate so that it did not cover the sample surface. The system was incubated in aerobic conditions at 37 °C for 24 h. After incubation, 10 μL of bacteria suspension from the surface of each sample was collected and seeded on a solid LB plate in Petri dishes. Then these solid LB plates were incubated in anaerobic conditions at 37 °C for another 24 h. After that, CFUs were counted using optical microscopes (Olympus BX63, Tokyo, Japan) and ImageJ software (1.53t version) [56]. As for the second CFU estimation method, bacteria concentration for the same samples was also determined spectrophotometrically on the basis of *E. coli* calibration curves. This estimation was conducted using solution optical density (OD) value as the comparative parameter. For this, bacteria suspensions after incubation on the sample surfaces were placed in 96-well plates and OD was measured at λ = 600 nm [57].

## 3. Results and Discussion

### 3.1. Materials Structure

**Figure 1:**
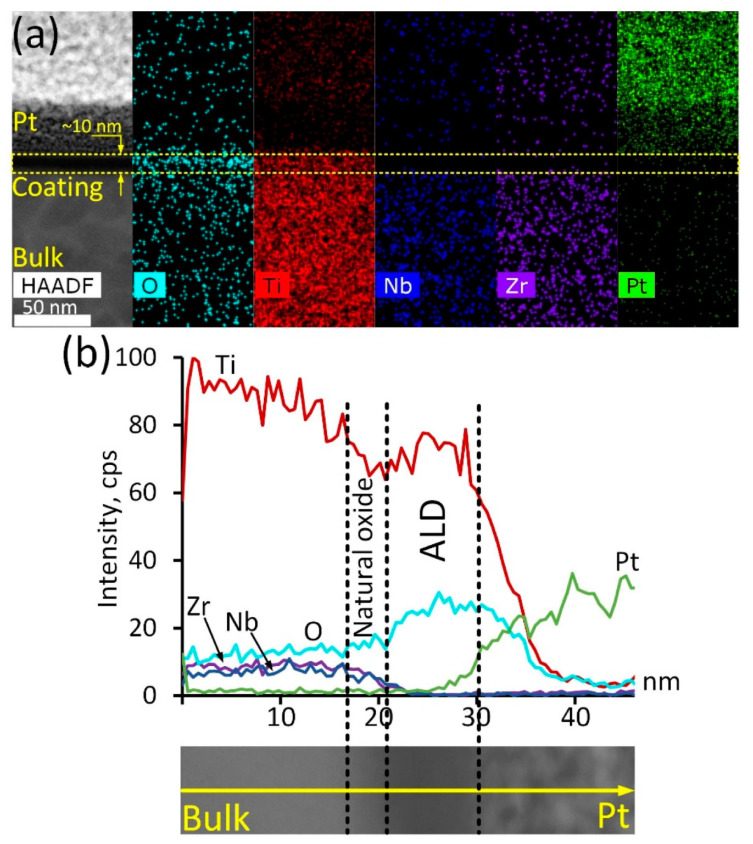
ALD TiO_2_ coating cross-section structure: HAADF electron image and corresponding elemental maps (**a**), elemental profile and corresponding HAADF electron image (**b**).

In-depth analysis of the ALD TiO_2_ coating structure and composition was conducted via TEM studies (Figure 1). The ALD coating is represented by a thin (10 nm) oxide layer, which is vividly seen in the HAADF electron image (Figure 1a). Corresponding elemental maps demonstrate that this layer is enriched with oxygen and contains significant amounts of titanium. It should be noted that this layer is completely free from Zr and Nb, proving that the formed oxide layer consists of Ti oxide exclusively. Note that the Pt signal is due to the platinum layer formed during the lamella preparation. Elemental profiling of a smaller region of the cross-section surface (Figure 1b) reveals other peculiarities. It can be seen that Ti concentration decreases gradually as the analysis spot shifts from the bulk alloy towards the coating region. The oxygen content increases slowly, while the Nb/Zr concentration remains at the same level. At a certain point, the concentration of the main alloy components decreases rapidly: Ti concentration drops by 10–15%, while Zr and Nb contents decrease to zero. In contrast, the oxygen concentration increases significantly. It can be assumed that this thin region of approximately 2–4 nm in thickness is a natural oxide film of the alloy, containing all its components. Closer to the surface, Ti and O concentrations rise rapidly, which is associated with the appearance of the TiO_2_ ALD coating. According to the STEM image, the width of this region is about 10 nm, which correlates well with the results reported in [35]. On the other hand, considering Ti and O concentration profiles, it can be assumed that the coating is thicker—up to 18 nm.

**Figure 2:**
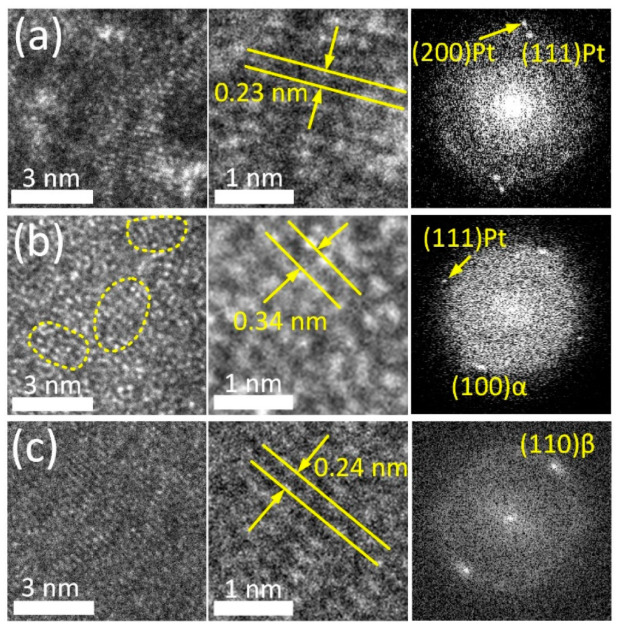
HRTEM images and FFT patterns of Pt layer (**a**), coating region (**b**) and adjacent bulk alloy (**c**).

The crystal structure of the TiO_2_ ALD coating was studied in more detail based on high-resolution TEM (HRTEM) electron images and the corresponding FFT diffraction patterns (Figure 2).

HRTEM images allow clear observation of individual atomic arrangements in the bulk alloy and the supportive Pt layer (Figure 2a,c). FFT patterns of these regions reveal the reflexes of the parent β-phase (110) with an interplanar spacing of 0.239 nm and metallic Pt (111) and (200) with an interplanar spacing of 0.227 nm and 0.197 nm, respectively. Conversely, the HRTEM image of the ALD coating (Figure 2b) demonstrates amorphous-like structure containing numerous ordered nanodomains in the amorphous matrix (outlined by a dashed line in Figure 2b). Similar types of microstructure were reported for severely deformed Ti-Ni and Ti-Zr-Nb SMAs [58,59]. The interplanar distance in ordered regions is ~0.34 nm. This value corresponds to the characteristic interplanar spacing of TiO_2_ (anatase modification) with *d* of 0.352 nm. The FFT pattern obtained from this region reveals a broad diffused ring and point reflections from the bulk alloy and Pt layer. This finding supports the suggestion that the ALD coating has a low degree of crystallinity. Note that in the alloy, at the boundary with the ALD coating, an α-phase (100) with d 0.256 nm is observed. Enhanced oxygen content in the subsurface layer stabilizes α-phase and its solubility in this phase reaches 30% as compared to ~8% in the parent β-phase. This factor apparently promotes β → α-phase transformation in the near-surface region during the ALD process [26,60,61].

**Figure 3:**
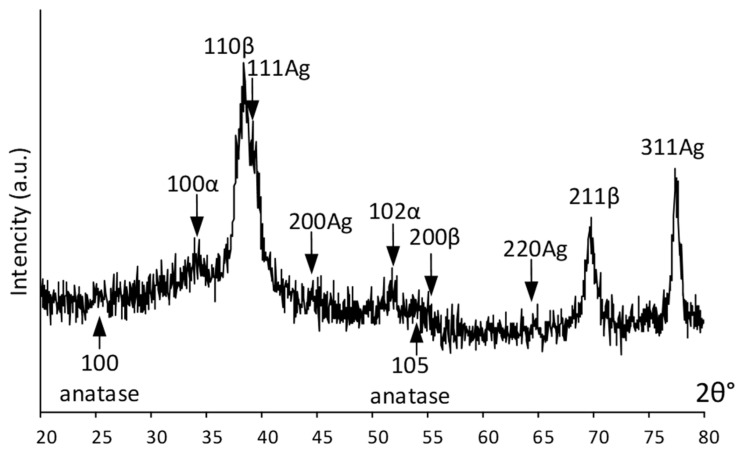
XRD pattern of Ag(d)/ALD sample.

An integral estimation of the surface and near-surface phase composition was obtained using X-ray diffraction. Figure 3 shows the X-ray pattern of the Ag(d)/ALD sample. It can be seen that the signal from the bulk alloy is quite pronounced and the {110} and {211} diffraction lines of the parent β-phase from the near-surface layer are distinctly visible in Figure 3. Low-intensity signals from the TiO_2_ coating (weak {100} and {105} anatase lines) obtained via the ALD process indicate its rather low thickness. Relatively strong {111}, {311} and weak {200}, {220} Ag reflections indicate significant amounts on the surface. Distinct peaks at ~35° and 52° 2θ deg are attributed to a low-temperature α-phase, which correlates well with the HRTEM results.

Summarizing the XRD and HRTEM results, it can be highlighted that the coating is in the amorphized state. Such a low crystallinity of the coating can be attributed to the selected ALD regime. The coating formation process is cyclic. With a small number of cycles, the coating thickness and crystallinity are low and with an increase in the number of cycles, the coating becomes thicker and more ordered.

**Figure 4:**
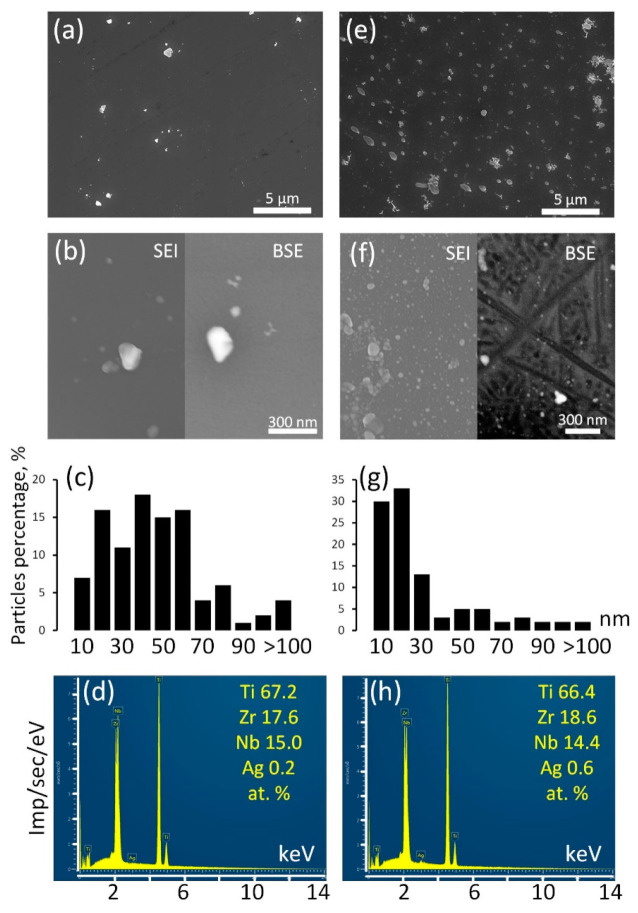
SEM images in SEI and BSE modes (**a**,**b**,**e**,**f**), Ag NP size distribution (**c**,**g**), EDX spectra (**d**,**h**) for Ag/ALD (**a**–**d**) and Ag(d)/ALD (**e**–**h**) samples.

Surface structure of the Ag NP-decorated substrates was analyzed by SEM and EDX (Figure 4).

The surface of the Ag/ALD sample is decorated with relatively rare Ag NPs (Figure 4a,b). Their sizes vary in the range from a few nm to a submicron scale. According to the size distribution diagram (Figure 4c), the majority of the Ag NPs have sizes from 40–60 nm. EDX analysis reveals that integral Ag concentration is approximately 0.2 at.%. It can be suggested that colloidal Ag NPs have poor affinity for TiO_2_ coating formed during ALD. In the Ag(d)/ALD sample, the structure is different. The SEM image obtained at a lower magnification (Figure 4e) demonstrates the presence of a significant amount of submicron Ag NPs evenly distributed over the TiO_2_ coating. Thorough investigation at higher magnification reveals numerous nanosized Ag NPs densely populating the sample surface (Figure 4f). It should be noted that in the BSE mode, small NPs disappear from the image, which indicates that they reside on the sample surface and are not incorporated into the coating. Ag NPs obtained by this method are much more monodisperse than in the Ag/ALD sample; the vast majority of NPs are smaller than 20 nm (Figure 4g). As expected, the Ag concentration increases as well and reaches 0.6 at.% (Figure 4h). Thus, the deposition of Ag NPs from a colloid solution over the ALD coating appears to be less efficient than the proposed modified technique, which provides a more even NP distribution and higher NP concentration.

### 3.2. Surface Chemical State

**Figure 5:**
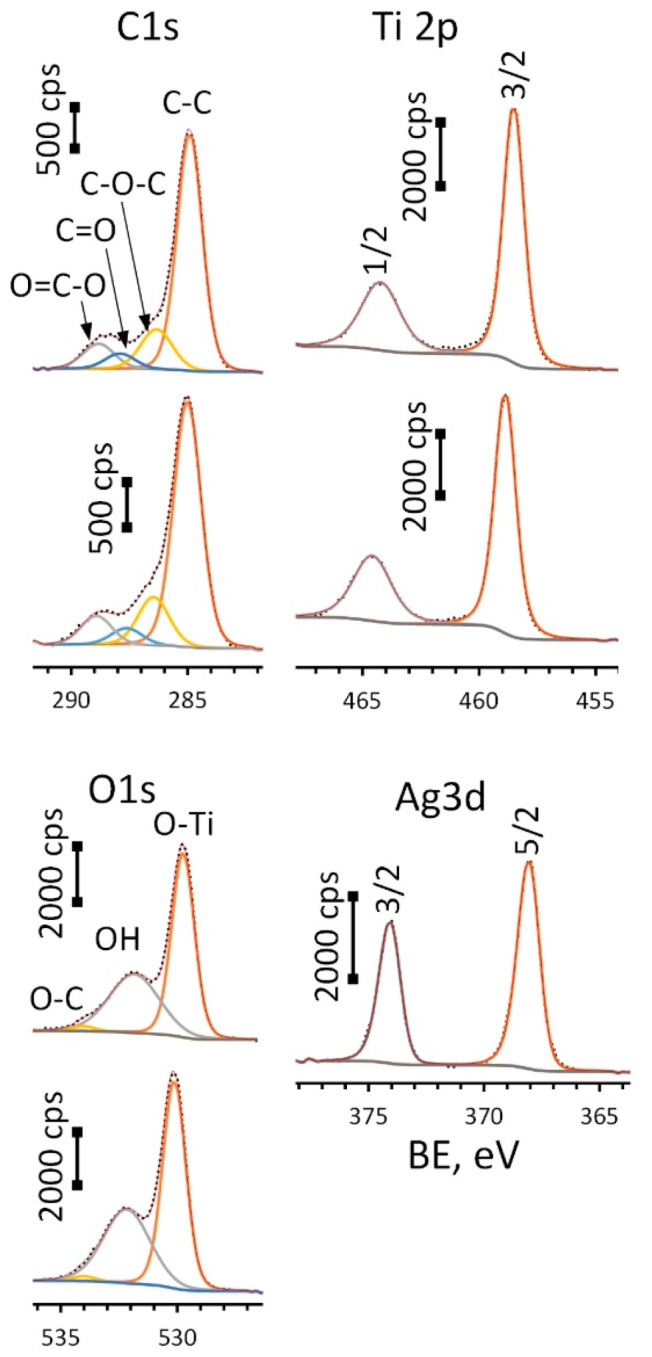
HR-XPS spectra for C1s, O1s, Ti2p (upper spectrum in each pair is for ALD and lower—Ag(d)/ALD samples) and Ag 3d peaks (Ag(d)/ALD sample).

High-resolution (HR) XPS spectra for Ag-free and Ag-decorated substrates are depicted in Figure 5.

Spectra for oxygen (O1s) were deconvoluted into three components: O-Ti bond located at 529.8 eV (ALD) and 530.1 eV (Ag(d)/ALD), surface hydroxyl groups—532.0 ± 0.2 eV and small amount of O-C species—534.0 ± 0.2 eV. A Ti2p doublet was fitted using two components: Ti2p 3/2 located at 464.2 eV for ALD and 464.6 eV for Ag(d)/ALD and Ti2p 1/2 located at 458.5 eV for ALD and 458.9 eV for Ag(d)/ALD. The spin-orbital gap ΔE value for these components remains the same for the given spectra and equals 5.7 eV, which is typical for titanium oxide (IV). What is more interesting is the significant shift of the Ti2p peak towards higher BE values observed for Ag-decorated substrate as compared to the Ag-free one. This shift of 0.4 eV can be associated with charge transfer from the oxide to Ag NPs. This means that the interaction between TiO_2_ film and Ag NPs can lead to the formation of a chemical bond that promotes NP fixation. The Ag3d doublet was deconvoluted into two peaks: Ag3d 5/2 and 3/2 located at 374.0 eV and 368.0 eV, respectively, well approximated by asymmetric function typical for Ag^0^. Additionally, the metallic nature of the silver is confirmed by ΔE value of 6.0 eV. The presence of carbon is associated with the organic contaminants at the substrate surface. Table 1 shows the surface chemical composition of the studied materials before and after Ar^+^ sputtering.

**Figure 6:**
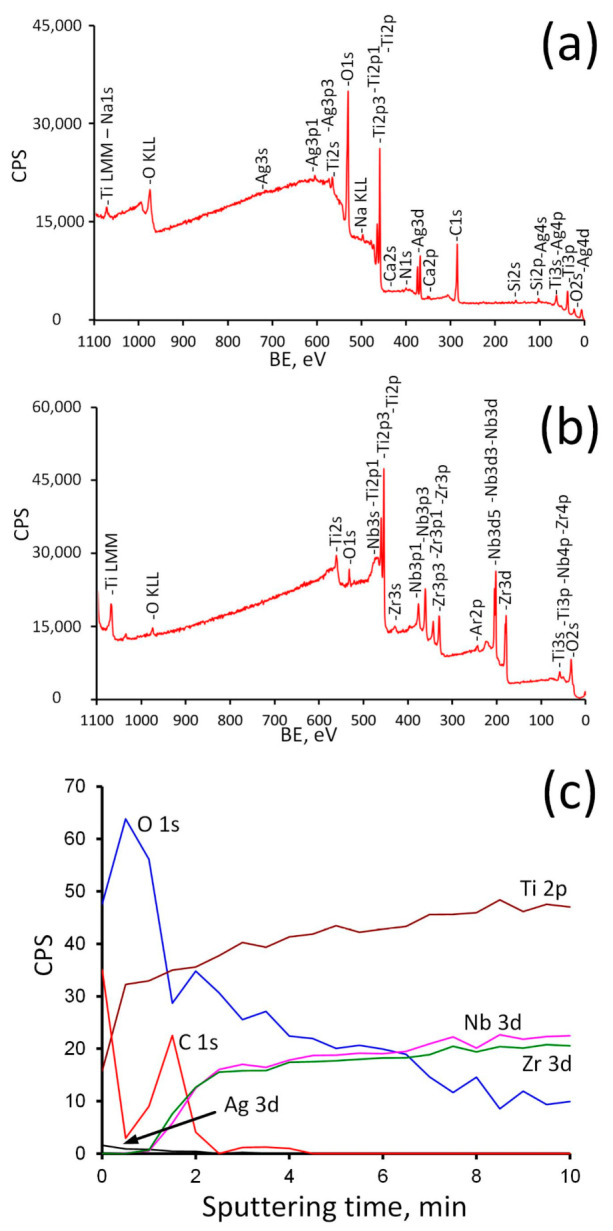
Survey spectra before (**a**) and after (**b**) Ar^+^ sputtering and depth profile (**c**) for Ag(d)/ALD sample.

It can be seen that the topmost sample layer consists only of titanium oxide. It indicates that the ALD coating was successfully formed on the surface of Ti-18Zr-15Nb alloy. The natural oxide layer contains all main components of the alloy [62], in our case Zr and Nb. The silver content in the Ag/ALD sample is quite low and noticeably higher for Ag(d)/ALD, which is in a good agreement with the EDX results. After Ar^+^ sputtering for 10 min, the formed coating as well as surface contaminants are removed and the alloy subsurface layer is exposed. A small amount of oxygen is still present, which indicates that subsurface layers contain some amount of suboxides, and the ratio between the main components differs from that in the alloy. Element depth profile for the Ag(d)/ALD sample is shown in Figure 6. Excess of oxygen and carbon as well as a negligible amount of N, Si and Ca are attributed to the surface contamination of the sample after the coating deposition process.

As follows from the analysis of the survey spectra, the signals from Ag3d completely disappear after Ar+ sputtering, while the peaks of Nb and Zr, on the contrary, become noticeable. The concentration profile shows that oxygen content decreases rapidly during the first 1.5 min of sputtering (approximately 13 nm of surface layer is removed). It can be assumed that during this time the titanium oxide coating is removed completely and suboxides layer is exposed. Then the oxygen content decreases gradually and reaches a plateau after 7 min of sputtering. At the same time, the concentration of Zr and Nb rises sharply after 1–1.5 min of sputtering, thereby indicating that the ALD coating was removed. A further increase in their concentration reflects a decrease in the content of suboxides (Zr^2+^, Zr^3+^, Nb^5+^, Nb^4+^) with distance from the surface. Ag content drops to zero after 2 min of sputtering (~17 nm depth). This means that Ag NPs reside on the surface and are not incorporated into the ALD film (Figure 4).

**Figure 7:**
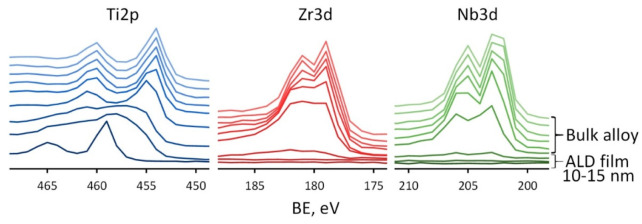
XPS spectra for Ti2p, Zr3d, Nb3d recorded at various sputtering times for Ag(d)/ALD sample.

The change in composition upon passing from the ALD TiO_2_ film to the bulk alloy is clearly seen in a series of XPS spectra for Ti, Zr and Nb, recorded at various Ar^+^ sputtering times (Figure 7).

Each profile is recorded after 30 s of Ar^+^ sputtering of a previous layer. The transition from TiO_2_ film to the bulk alloy is seen as a large shift of Ti2p3/2 peak from ~459 eV corresponding to Ti^4+^ to ~454 eV corresponding to Ti^0^ observed after 1.5–2 min of sputtering, i.e., at the depth of ~13–17 nm, which corresponds well to the concentration depth profile. In the case of Zr and Nb the results are different. Signals from these elements appear immediately after the removal of the TiO_2_ film, which confirms their presence only in the substrate. After 1.5 min of sputtering, the signals from the Zr and Nb are weakly expressed, but as soon as two more layers are removed, well-defined doublets of Zr3d and Nb3d are observed with the position of Zr3d 5/2 at 179.0 eV and Nb3d 5/2 at 202.0 eV, which are typical for Zr^0^ and Nb^0^. Thus, the proposed ALD method makes it possible to avoid the formation of alloying elements oxides on the material’s surface. This result can be very useful for further surface modification of Ti-Zr-Nb alloys. Indeed, the natural oxide layer of this alloy is quite complex and contains a mixture of oxides of various elements present in the alloy. Modification of its surface, for example, by chemical etching, can be quite complex, since all oxides have different chemical stability. Therefore, the controlled-thickness TiO_2_ film ALD technology is much more attractive.

### 3.3. Antibacterial Properties

**Figure 8:**
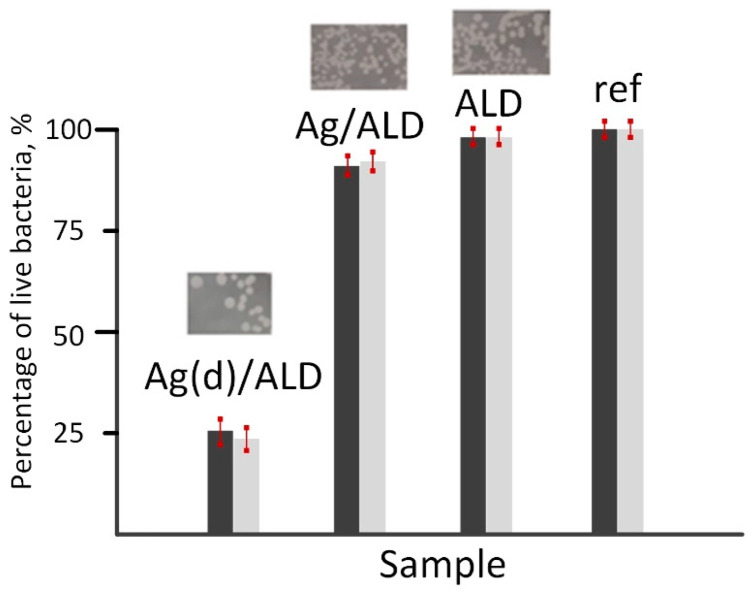
Bacterial growth inhibition on the surface of Ag(d)/ALD, Ag/ALD, ALD and reference samples. Dark bars—optical microscopy results, light bars—spectrophotometry results.

The antibacterial properties of metal alloys were evaluated based on the results of the inhibition of bacterial growth on the sample surfaces after 24 h of incubation. Two methods were used: plate count testing and optical density measurement. The obtained results were combined into the final statistics. The results of the antibacterial tests are collected in Figure 8.

It should be noted that the results of both methods correlate well with each other. The highest antibacterial activity with more than 75% inhibition of *E. coli* strain is observed for the Ag(d)/ALD sample. The Ag/ALD material demonstrates a much less pronounced effect with only 10% growth inhibition. The ALD sample showed almost identical results to the control. It can be concluded that the antibacterial effect of the studied materials is due solely to the presence of Ag species on their surfaces. Analysis of the release of Ag ions from the sample surfaces showed that their concentration is 0.0268 mg/L (Ag(d)/ALD) and 0.0089 mg/L (Ag/ALD). This explains the difference in antibacterial activity. Destruction of the *E. coli* cell wall occurs more easily with a higher concentration of Ag^+^ species [63]. The weaker antibacterial effect in the ALD sample can be associated with the formation of a smoother oxide surface after ALD, which is unfavorable for bacterial cell adhesion compared to the polished alloy surface.

Surface modification of superelastic Ti-Zr-Nb alloys by ALD of a thin titanium oxide film and subsequent precipitation of Ag nanoparticles are of great importance for the wider application of these materials in medicine. This approach can be used to modify complex surfaces with developed roughness, such as highly porous implants obtained by additive technologies. A more careful selection of ALD parameters for Ti-Zr-Nb alloys will make it possible to form a more complex surface topology. For example, the ALD method can be used to form TiO_2_ nanotubes and nanoflakes, which potentially have a more pronounced bioactivity and enhanced osseointegration. This can solve the well-known problem of the presence of bioinert Ti, Zr and Nb oxides on the surface of Ti-Zr-Nb implants designed to replace bone tissue. The presence of surface Ag nanoparticles can prevent or suppress infection, the threat of which is at its maximum in the first days after bone replacement. Thus, our results make it possible to expand the possibilities of using Ti-Zr-Nb superelastic alloys for bone tissue replacement.

## 4. Conclusions

In this work, a novel method of superelastic Ti-18Zr-15Nb alloy surface modification was developed. Atomic layer deposition (ALD) technique was implemented in order to obtain a thin amorphized TiO_2_ film with a controlled thickness over the natural alloy oxide film. The near-surface layers of the alloy were enriched with oxygen and contained the α-phase, while the β-phase was present further from the surface. The XPS analysis showed that the ALD TiO_2_ film with a thickness of 10–15 nm does not contain oxides and suboxides of doping elements (Zr and Nb). Two methods for obtaining Ag NPs on the ALD film surface were used and compared. The precipitation of Ag NPs from the colloidal solution resulted in a low content of Ag on the surface, which is associated with the low affinity of Ag NPs for the smooth TiO_2_ film. Pretreatment of the Ti-18Zr-15Nb alloy in NaBH_4_ solution prior to colloidal treatment made it possible to significantly increase the concentration of Ag NPs on the surface. Ag NPs were uniformly distributed over the surface of the modified alloy and their size did not exceed 30 nm. The material with a higher Ag content showed a stronger antibacterial effect against *E. coli* bacteria (75% of the cells were inactivated) compared to 10% for the material with a lower Ag content. The developed method of two-stage modification can be used to control the surface chemical state and the antibacterial properties of new superelastic biomedical alloys.

## Figures and Tables

**Table 1:** Surface chemical composition, at.%.

Sample	C	O	Ti	Zr	Nb	Ag	N	Si	Ca
ALD	38.3	46.4	13.0	-	-	-	0.9	0.8	0.5
Ag/ALD	39.8	44.8	12.8	-	-	0.4	1.0	1.1	0.1
Ag(d)/ALD	37.7	45.4	12.3	-	-	1.6	1.0	1.7	0.3
Ar^+^ sputtering									
ALD	-	12.4	47.8	18.7	21.1	-	-	-	-
Ag(d)/ALD	-	11.6	48.3	19.2	20.9	-	-	-	-

## Data Availability

Not applicable.
